# Leisure activities of adolescents—associations with demographic characteristics, well-being and parental leisure engagement

**DOI:** 10.1038/s41390-025-03866-9

**Published:** 2025-01-23

**Authors:** Friederike Wanka, Mandy Vogel, Nico Grafe, Manuela Aßmann, Wieland Kiess, Tanja Poulain

**Affiliations:** 1 LIFE Leipzig Research Center for Civilization Diseases, Leipzig University, Leipzig, Germany; 2 Department of Women and Child Health, Hospital for Children and Adolescents and Center for Pediatric Research (CPL), Leipzig University, Leipzig, Germany

## Abstract

**Background:**

The present study aimed to investigate associations between leisure activities and well-being, behavioral difficulties, and parental leisure time engagement.

**Methods:**

A total of 959 adolescents aged 10 to 18 years were included in the present study. We assessed their leisure activities (e.g., family time, meeting friends, screen time, sports participation), well-being (Kidscreen-27) and behavioral difficulties (Strengths and Difficulties Questionnaire SDQ) using questionnaires. The leisure behavior of parents was assessed using a parent version of the leisure activities questionnaire. We applied linear and logistic mixed-effect models to assess associations, adjusting for age, sex, and SES.

**Results:**

The associations between leisure activities and well-being differed depending on the type of activity. Active leisure (e.g., family time, meeting friends, café visits, visiting a museum/art exhibition/cinema, visiting a concert/opera, voluntary work, sports participation) was associated with better well-being, especially physical well-being, and fewer behavioral difficulties. Passive leisure activities (screen time, chilling) were associated with lower well-being and more behavioral difficulties. We also observed that adolescents reported more frequently participation in the described leisure activities if their parents did so.

**Conclusion:**

We need to promote active leisure behavior among young people and their parents to improve adolescents’ well-being.

**Impact:**

- Most previous studies on leisure behavior have focused on screen time and sports participation, but we examined a variety of leisure activities including family time, meeting friends, café visits, visiting a museum/art exhibition/cinema, visiting a concert/opera, voluntary work, chilling and sports participation and screen time.
- Active leisure activities are associated with better well-being and less behavioral problems.
- Parents’ leisure time participation is associated with higher participation of their children in these activities.
- We need to promote active leisure behavior among adolescents and their parents to improve overall well-being.

## Introduction

Adolescence, usually defined as the age period between 10 and 25 years,^1^ is a critical developmental period in which individuals become increasingly independent from their parents. Consequently, their leisure behavior becomes more self-determined during adolescence. Important leisure activities for adolescents are extracurricular activities, physical activity and media use, although only the latter two have been analyzed in more detail in recent decades.

Physical activity is a key factor in preventing the development of chronic diseases such as obesity and its comorbidities.^2^ Moreover, it is also associated with a lower risk for anxiety and depression and thus promotes mental health.^2,3^ For children and adolescents aged five to 17 years, the World Health Organization (WHO) recommends an average of 60 min of moderate to vigorous physical activity (e.g., walking, running) per day.^4^ In addition, the WHO suggests incorporating vigorous-intensity aerobic activity and bone-strengthening, i.e., climbing, jumping, or running, at least three days a week.^4^

Media use, another common leisure activity, is a type of sedentary behavior that includes the use of television, radio, video games, mobile phones and the internet.^5^ Access to information or contact with friends and family are some observed benefits.^6^ However, media use is also associated with an increased risk of cyberbullying and problems with online safety.^5^ In addition, intensive usage of screen-based media has been associated with more sleep difficulties,^7^ behavioral difficulties, and decreased psychological well-being.^8^ Moreover, associations with lower academic performance, lower physical fitness^9^ and higher obesity rates were observed.^10–12^

Studies on leisure activities other than physical activity and media use are sparse. The few existing studies show that extracurricular activities (including not only sports participation but also performing arts, participation in academic clubs and student government) are associated with the development of social and physical skills, improvement of school performance and interpersonal competence,^13^ as well as lower rates of depression^14^ and improved mental health.^15^ Family time is also negatively associated with depression^16^ and possibly mediated by better family functioning, better emotional health and improved cognitive abilities.^17^

Examining how adolescents spend their leisure time requires considering certain factors such as age, sex and socioeconomic status (SES) as these factors might impact leisure behavior.^18–21^ Additionally, parents’ leisure time involvement may also influence the leisure behavior of their children. Previous studies showed that boys are significantly more active than girls, but the latter participate more frequently in activities such as choir, orchestra or theatre.^19^ In addition, higher SES is associated with more physical activity^18,19^ and less screen time.^19,20^ Older adolescents are less active than younger adolescents, and also report more screen time.^19,20^ Regarding the potential impact of parents’ leisure behavior, previous studies have shown that parental physical activity and media use are associated with the frequency of these activities in their children.^22,23^

This study pursues several objectives. First, we investigated how various factors such as age, sex, SES and parental involvement in leisure time affect the leisure behavior (sports participation, screen time, and other) of adolescents. We then analyzed the relationship between the above-mentioned activities and the subjective well-being of adolescents. Based on previous research, we expected that parents’ leisure activities would be associated with a higher frequency of these activities in their children. We also expected that extracurricular activities and sports participation would be positively associated with higher well-being, especially higher physical and psychological well-being.

## Methods

### Study design

The present project was realized between 2017 and 2021 within the LIFE Child study situated at the Leipzig Research Center for Civilization Diseases (Germany). The childhood cohort study is designed to assess healthy child development and to understand the interplay of various internal (biological, genetic) and external (social environment, social class, lifestyle) factors in shaping children’s and adolescents’ well-being.^24,25^ Within the large LIFE Child project, children and adolescents not suffering from any chronic, chromosomal or syndromal diseases are recruited until the age of 16 years, mainly via advertisements in hospitals, clinics, schools, and media, and by word of mouth. They are invited to attend follow-up visits until the age of 21 years. Depending on child age, the study program includes different interviews, a medical examination, questionnaires, and the collection of blood samples.

The LIFE Child study was designed in compliance with the Declaration of Helsinki and was approved by the Ethical Committee of the University of Leipzig. Written informed consent of all parents and of adolescents aged 12 years and above was provided at each study visit.^24,25^

For the present project, all adolescents who had completed the questionnaire on leisure activity were eligible for analysis. This precondition was met by 959 adolescents (496 male, 463 female) aged 10 to 18 years. If an adolescent had participated in the study at more than one-time point (*n* = 949), only the last visit was considered. The mean age in the final sample was 14.2 (SD = 2.14). For further analysis, we created two age groups, separated by median split; a group of young adolescents aged 10 to 13 years (*n* = 407, 51.7% male, mean age = 12.0) and a group of old adolescents aged 14 to 18 years (*n* = 552, 52.2% male, mean age = 15.8). Of the 959 adolescents, not all answered questions on media use/reading (*n* = 618, 52.75% male, 35.76% young age group, mean age = 14.5) and sports participation (*n* = 924, 51.73% male, 41.77% young age group, mean age = 14.2), leading to smaller samples in the according analyses. The characteristics of the study population are shown in Table 1.

**Table 1:** Descriptive statistics in the present sample (*N* = 959).

Independent variables			Total number
Sex	Male	*N* (%)	496 (51.7%)
	Female	*N* (%)	463 (48.3%)
Age Group	10–13 years	*N* (%)	407 (42.4%)
	14–18 years	*N* (%)	552 (57.6%)
SES	Low	*N* (%)	37 (3.9%)
	Medium	*N* (%)	547 (57.0%)
	Low/Medium	*N* (%)	584 (60.9%)
	High	*N* (%)	375 (39.1%)
Dependent Variables			
Kidscreen-27^a^	Physical well-being	*M* (SD)	51.31 (10.12)
	Psychological well-being	*M* (SD)	50.38 (10.06)
	Parent relation/autonomy	*M* (SD)	55.57 (10.10)
	Peers/social support	*M* (SD)	52.95 (10.53)
	School environment	*M* (SD)	53.13 (9.95)
SDQ	Total difficulties score	*M* (SD)	10.04 (5.14)

*N* total number, *M* mean, *SD* standard deviation.

^a^*t*-values based on sex- and age-specific references (*M* = 50, SD = 10).

### Questionnaires

Data analyzed in the present study were collected via questionnaires. While questionnaires on well-being, behavioral strengths and difficulties, and SES were standard questionnaires or designed for another study, all other questionnaires were developed by the LIFE Child study research team. Questionnaires regarding the adolescent’s behavior were completed by the participants themselves. Questions concerning the family’s SES and the leisure activity of parents were completed by the participants’ parents.

#### Leisure time of adolescents and their parents

Adolescents themselves and their parents answered the same questions on their leisure behavior. They were asked to indicate how much time they spend on different leisure activities. The activities recorded included time with family, meeting friends, chilling, café/restaurant visits, going to museum/cinema/art exhibition, going to concert/opera, participation in choir/orchestra/theater, and voluntary work. The participants were asked to choose between six answer options for each activity (1-never, 2-less than once a year, 3-once a year, 4-once a month, 5-once a week, and 6-daily). For each activity, responses were dichotomized using a median split. For time with family, meeting friends, and chilling, we contrasted the categories “less than once a week” (response options 1–4) and “minimum of once a week” (response options 5–6). For café/restaurant visits and visiting a museum/art exhibition/cinema, we created the categories “less than once a month” (response options 1–3) and “minimum of once a month” (response options 4–6). For visiting a concert/opera, we contrasted the categories “never” (response option 1) and “minimum of once a year” (response options 2–6). For choir/orchestra/theater, we compared the categories “no participation” (response option 1) and “participation” (response options 2–6). Voluntary work included involvement in school, church, politics, and social services. Adolescents could indicate whether they volunteered in any of the abovementioned areas; therefore distinguished between “participation” and “no participation”.

The leisure behavior of parents was assessed using a parent version of the abovementioned questionnaire. The categorization of item responses was the same as in adolescents.

#### Media use and reading of adolescents and parents

Adolescents and parents answered the same questions on their media use and reading behavior. In the questionnaire on media use, they were asked to indicate how much time they spend with different media devices (computer, smartphone, video console, tablet, television) and with reading on a normal weekday and on a weekend day. For each question, they could choose between five response options (none, 30 min per day, 1–2 h per day, 3–4 h per day, and more than 4 h per day). The responses were transformed into numeric durations per day (none → 0 h, 30 min →0.5 h, 1–2 h →1.5 h, 3–4 h → 3.5 h, more than 4 h→5 h). Subsequently, the weighted (daily) means per medium were summed up to the variable “daily screen time” (daily screen time = (duration computer on weekday*5+duration computer weekend*2)/7 + (duration smartphone on weekday*5+duration smartphone weekend*2)/7 + (duration video console on weekday*5+duration video console weekend*2)/7 + (duration tablet on weekday*5+duration tablet weekend*2)/7). We applied a median split and contrasted the categories “less than 5 h a day” and “a minimum of 5 h a day” for adolescents and parents.

After performing a median split for the reading durations per day, we contrasted the response categories “≤30 min/day” and “>30 min/day” for both adolescents and parents.

#### Sports participation

In the sports participation questionnaire, adolescents and parents were asked whether they participated in any organized sports. This dichotomous variable (participation versus no participation) was used for further analysis.

#### Well-being

Participants’ well-being was assessed using the KIDSCREEN-27 questionnaire. It comprises a total of 27 items measuring five dimensions of well-being: physical well-being (5 items), psychological well-being (7 items), autonomy (7 items), peers and social support (4 items), and school environment (4 items).^26^ All questions are answered on a five-point Likert scale. For each dimension, the item scores of the different questions are summed, with higher sum scores indicating higher well-being. The sum scores are transformed to *t*-values, based on sex- and age-specific references (*M* = 50, SD = 10). These *t*-values were used for further analysis. Internal consistency was assessed using Cronbach’s alpha for each dimension of the Kidscreen-27 and ranged from 0.79 to 0.85, suggesting strong correlations among variables of the same scale. Validity of the questionnaire has been shown in a previous study.^26^

#### SDQ

The Strengths and Difficulties Questionnaire (SDQ) is an assessment tool including 25 items on four difficulty scales (emotional difficulties, behavioral difficulties, hyperactivity/inattention, peer difficulties) and one scale on behavioral strengths (prosocial behavior). Each scale includes five items with three response options, namely “not true” (0), “somewhat true” (1), and “certainly true” (2). Each score ranged from 0 to 10. The four difficulty scales can be combined into a total difficulty score ranging from 0 to 40, with higher scores indicating greater behavior difficulties.^27^ This score was used for further analysis. For all items included in the difficulties score, Cronbach’s alpha was 0.72, demonstrating a good reliability. The validity of the questionnaire has been shown in other studies.^28,29^

#### SES

The SES of participants was assessed by considering the highest education, occupation, and monthly household income of the participant’s parents. Information on these aspects was combined into a SES composite score.^30^ The score ranges from 3 to 21, with higher scores reflecting higher SES. Using cutoffs based on data collected in a representative German sample, the score can be classified into low (3–8.4 points), middle (8.5–15.4 points), or high (15.5–21) SES.^31^ In a representative sample, the distribution of low, middle, and high SES is expected to be 20%, 60%, and 20%. In the present study, 3.9% belonged to the low, 57.0% to the middle and 39.1% to the highest SES group. Due to the low number of participants from low SES families, we combined low and middle SES into one category (low/medium), which then included 60.9% of the families.

### Statistical analysis

All analyses were performed using R version 4.0. The first analysis, a multiple logistic mixed-effect model, investigated associations between each leisure activity (as the dependent variable) and sex, age group, and SES (as independent variables, included simultaneously).

In the second step, we applied linear mixed-effect models (including the family ID as a random effect) to examine the relationship between each leisure activity (as independent variables) and the different dimensions of well-being and the total behavioral difficulties score (as dependent variables).

In a third step, we used logistic mixed-effect models to assess associations between parental leisure activities (as the independent variable) and leisure activities of their children (as the dependent variable).

Strengths of associations were reported as odds ratios (in the case of logistic regressions) or nonstandardized coefficients (in the case of linear regressions). The level of significance was set to *α* = 0.05. To adjust the p-values for multiple comparisons, we applied the Benjamini and Hochberg correction. This correction resulted in a loss of significance for six associations which are highlighted in the corresponding tables. As age, sex, and SES may influence well-being, leisure behavior and, therefore, the association between the two, they were included as covariates in all models. Furthermore, sibling relationships within the sample were accounted for by including the family ID as a random effect. The family ID is an additional pseudonym, which, in contrast to the individual ID, is the same for all members of the same family (including stepfamily members).

## Results

**Fig. 1:**
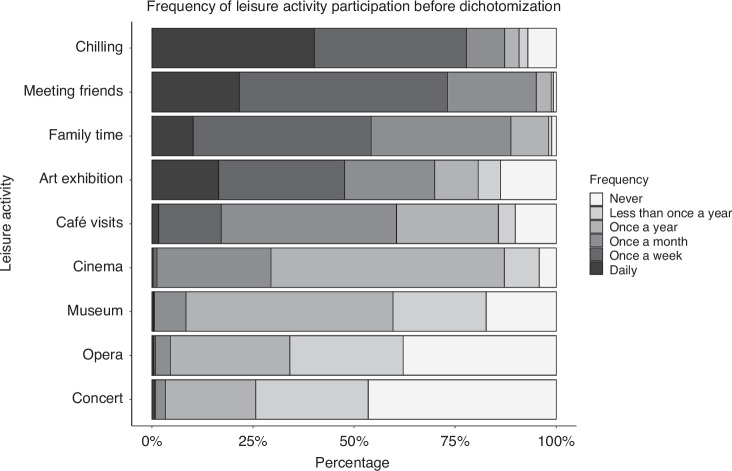
Depicts the frequency of selected leisure activities (all but media use and sports participation) before dichotomization. The filling of the bars represents the possible frequencies of participation. The shading within the bars ranges from dark (daily) to white (never). Chilling was the most frequently reported activity, followed by meeting friends. Less common activities were going to the cinema, museum or art exhibition as well as going to a concert or opera.

The characteristics of the study population are shown in Table 1. Frequencies of girls and boys and of both age groups were comparable. The distribution of the total difficulties and Kidscreen-27 score were not normally distributed. While the total difficulties score was right skewed (many low values, few high values), as also indicated by a low mean value (10.04), the Kidscreen-27 scores were left skewed (few low values, many high values). Figure 1 presents the participation of adolescents in selected activities before dichotomization.

### Frequency of leisure behavior and differences depending on sex, age, and SES

Table 2 shows the distribution of all assessed leisure activities after dichotomization among healthy adolescents. Table 3 displays the associations of these activities with sex, age and SES.

**Table 2:** Distribution of leisure activities among healthy adolescents in the LIFE Child study cohort, stratified by sex, age group, and SES (*N* = 959).

Leisure activity		All	Sex			Age group		SES group	
		*N* = 959		Male	Female	10–13 years	14–18 years	Low/Medium	High
Family time^a^	*959*	520 (54.2%)		270 (54.4%)	250 (54.0%)	251 (61.7%)	269 (48.7%)	294 (50.3%)	226 (60.3%)
Meeting friends^a^	*959*	701 (73.1%)		358 (72.2%)	343 (72.2%)	293 (72.0%)	408 (73.9%)	430 (73.6%)	271 (72.3%)
Chilling^a^	*959*	746 (77.8%)		369 (74.4%)	377 (81.4%)	293 (72.0%)	453 (82.1%)	466 (79.8%)	280 (74.7%)
Café visits^b^	*959*	580 (60.5%)		311 (62.7%)	269 (58.1%)	218 (53.6%)	362 (65.6%)	333 (57.0%)	247 (65.9%)
Museum/Art exhibition/Cinema^b^	*959*	750 (78.2%)		348 (70.2%)	402 (86.8%)	330 (81.1%)	420 (76.1%)	447 (76.5%)	303 (80.8%)
Concert/Opera^c^	*959*	726 (75.7%)		352 (71.0%)	374 (80.8%)	298 (73.2%)	428 (77.5%)	416 (71.2%)	310 (82.7%)
Choir/Orchestra/Theater^d^	*959*	486 (50.7%)		210 (42.3%)	276 (59.6%)	224 (55.0%)	262 (47.5%)	258 (44.2%)	228 (60.8%)
Voluntary work^d^	*959*	394 (41.1%)		190 (38.3%)	204 (44.1%)	177 (43.5%)	217 (39.3%)	222 (38.0%)	172 (45.9%)
Reading^e^	*618*	458 (74.1%)		212 (65.0%)	246 (84.3%)	190 (86.0%)	268 (67.5%)	272 (68.5%)	186 (84.2%)
Screen time^f^	*618*	370 (59.9%)		206 (63.2%)	164 (56.2%)	107 (48.4%)	263 (66.3%)	278 (70.0%)	92 (41.6%)
Sports participation^d^	*924*	551 (59.6%)		296 (61.9%)	255 (57.2%)	226 (58.6%)	325 (60.4%)	301 (53.2%)	250 (69.8%)

*SES* socio-economic status.

^a^Minimum of once a week.

^b^Minimum of once a month.

^c^Minimum of once a year.

^d^Overall participation.

^e^More than 30 min per day.

^f^More than 5 h per day.

**Table 3:** Associations between leisure activities and sex, age, and SES (*N* = 959): Results of logistic mixed-effect models.

Leisure activity	Sex (reference: male)	Age (reference: 10–13 years)	SES (reference: high SES)
	OR (95% CI)	OR (95% CI)	OR (95% CI)
			Low/Medium
Family time^a^	0.98 (0.74; 1.29)	0.59 (0.45; 0.79)**	0.68 (0.51; 0.91)**
Meeting friends^a^	1.11 (0.82; 1.50)	1.10 (0.81; 1.49)	1.07 (0.79; 1.46)
Chilling^a^	1.60 (1.12; 2.28)**	1.88 (1.32; 2.70)**	1.32 (0.92; 1.90)
Café visits^b^	0.81 (0.61; 1.08)	1.79 (1.34; 2.39)**	0.62 (0.46; 0.84)**
Museum/Art exhibition/Cinema^b^	2.97 (2.05; 4.31)**	0.74 (0.53; 1.05)	0.77 (0.54; 1.10)
Concert/Opera^c^	1.84 (1.31; 2.59)**	1.40 (1.00; 1.95)^x^	0.48 (0.33; 0.69)**
Choir/Orchestra/Theater^d^	2.17 (1.62; 2.90)**	0.76 (0.57; 1.01)	0.49 (0.36; 0.66)**
Voluntary work^d^	1.27 (0.98; 1.65)	0.87 (0.67; 1.13)	0.73 (0.56; 0.96)*
Reading^e^	3.26 (2.08; 5.08)**	0.32 (0.20; 0.51)**	0.40 (0.25; 0.64)**
Screen time^f^	0.68 (0.46; 1.00)^x^	2.20 (81.47; 3.31)**	3.78 (2.39; 5.98)**
Sports participation^d^	0.77 (0.57; 1.05)	1.20 (0.88; 1.63)	0.43 (0.31; 0.61)**

*SES* socioeconomic status, *OR* odds ratio, 95% *CI* 95% confidence interval.

*p < 0.05, **p < 0.01. x = statistically significant before, but not after correction for multiple testing.

^a^Minimum of once a week.

^b^Minimum of once a month.

^c^Minimum of once a year.

^d^Overall participation.

^e^More than 30 min per day.

^f^More than 5 h per day.

As can be seen, chilling (81.4% vs 74.4%), visiting a museum/art exhibition/cinema (86.8% vs 70.2%) and attending a concert/opera (80.8% vs 71.0%) were reported significantly more frequently by girls than boys (*p* < 0.01). In addition, girls reported engaging more frequently in choir/orchestra/theater (59.6% vs. 42.3%, *p* < 0.01) and reading (84.3% vs 65.0%) than boys.

The analyses also revealed significant age differences in leisure behavior. Chilling (82.1% vs. 72.0%), visiting a café/restaurant (65.6% vs 53.6%) and screen time (66.3% vs 48.4%) were reported significantly more frequently in old adolescents than in young adolescents. In contrast, older adolescents engaged significantly less frequently in family activities (48.7% vs. 61.7%) and reading (67.5% vs. 86.0%).

Regarding differences between SES groups, the analyses showed that adolescents of low/medium SES engaged significantly less frequently in family activities (50.3% vs. 60.4%), visiting cafés/restaurants (57.0% vs. 65.9%), attending a concert/opera (71.2% vs. 82.7%), choir/orchestra/theater (44.2% vs. 60.8%), voluntary work (38.0% vs. 45.9%), reading (68.5% vs. 84.2%), and sports (53.2% vs. 69.8%) than adolescents of high SES. In contrast, a low/medium SES was significantly associated with higher screen time, with 70.0% of respondents using electronic devices for more than 5 h a day, compared to 41.6% of high SES youth.

### Associations between leisure behavior and well-being

Leisure activities were significantly associated with the well-being of adolescents. The comprehensive results of the analyses are summarized in Table 4. Notably, spending time with the family at least once a week was significantly associated with higher well-being across all dimensions (*b* ranging between 1.79 and 3.05, all *p* < 0.01). Moreover, several other leisure activities showed positive associations with well-being in at least two domains. Meeting friends at least once a week was positively associated with physical well-being and more satisfaction with peer support (*b* ranging between 3.07 and 5.72, *p* < 0.01). Additionally, visiting cafés at least once a month was positively associated with higher physical well-being, better parent relation and enhanced peer support (*b* ranging between 1.76 and 2.61, all *p* < 0.01). Visiting a museum/art exhibition/cinema or a concert/opera and voluntary work were only associated with one or two dimensions of well-being. While attending a museum/art exhibition/cinema at least once a month was only associated with higher physical well-being (*b* = 3.46, *p* < 0.01), visiting a concert/opera at least once a year was associated with better peer support and school environment (*b* = 1.71 and 2.07, both *p* < 0.05). Additionally, positive associations were found between participation in voluntary work and increased physical well-being and satisfaction with school environment (*b* = 2.00 and 2.34, both *p* < 0.01). In contrast, chilling and a high level of screen time were associated with lower well-being in at least two domains. Chilling showed negative associations with psychological well-being and parent relation (*b* = –2.04 and –1.86, *p* < 0.05). Furthermore, participants who reported more than 5 h of daily media use experienced decreased physical, as well as psychological well-being along with decreased peer support (*b* ranging between –3.62 and –2.31, all *p* < 0.05). Finally, visiting choir/orchestra/theater and reading were the only activities that showed no significant association with any dimension of well-being.

**Table 4:** Associations between leisure activities and well-being among healthy adolescents in the LIFE Child study cohort (*N* = 959): Results of linear mixed-effect models.

Leisure activity	Kidscreen-27				
	*b*				
	(95% CI)				
	Physical well-being	Psychological well-being	Parent relation/ Autonomy	Peers/Social support	School environment
Family time^a^	3.05**	2.42**	2.84**	1.79**	2.10**
	1.80; 4.29	1.20; 3.64	1.56; 4.12	0.44; 3.14	0.84; 3.36
Meeting friends^a^	3.07**	1.49^x^	0.97	5.72**	1.54^x^
	1.69; 4.45	0.13; 2.85	–0.46; 2.40	4.26; 7.18	0.14; 2.93
Chilling^a^	–0.93	–2.04**	–1.86*	–0.17	–1.25
	–2.43; 0.58	–3.51; –0.57	–3.40; –0.32	–1.79; 1.46	–2.77; 0.26
Café visits^b^	2.10**	0.46	1.76**	2.61**	0.92
	0.82; 3.38	–0.80; 1.71	0.45; 3.08	1.23; 3.98	–0.37; 2.21
Museum/Art exhibition/Cinema^b^	3.46**	0.46	1.65^x^	1.25	0.78
	1.94; 4.98	–1.03; 1.96	0.08; 3.22	–0.40; 2.90	–0.76; 2.32
Concert/Opera^c^	1.46	0.16	0.70	2.07*	1.71*
	–0.01; 2.92	–1.27; 1.60	–0.81; 2.21	0.50; 3.65	0.24; 3.18
Choir/Orchestra/Theater^d^	–0.12	–0.50	0.88	–0.08	1.04
	–1.40; 1.16	–1.74; 0.75	–0.43; 2.19	–1.46; 1.30	–0.24; 2.33
Voluntary work^d^	2.00**	–0.14	0.17	1.03	2.34**
	0.75; 3.26	–1.38; 1.09	–1.12; 1.47	–0.33; 2.39	1.09; 3.60
Reading^e^	–0.31	–1.18	–1.18	–0.98	0.25
	–2.20; 1.58	–3.06; 0.70	–3.11; 0.74	–2.96; 0.99	–1.62; 2.11
Screen time^f^	–3.62**	–2.76*	–0.69	–2.31*	–2.42**
	–5.28; –1.95	–4.44; –1.09	–2.41; 1.04	–4.07; –0.55	–4.07; –0.77
Sports participation^d^	2.59**	0.21	–0.08	1.11	0.35
	1.29; 3.90	–1.08; 1.49	–1.44; 1.28	–0.31; 2.52	–0.98; 1.68

*b* non-standardized regression coefficient, 95% *CI* 95% confidence interval.

*p < 0.05, **p < 0.01, x = statistically significant before, but not after correction for multiple testing. All associations are adjusted for age, sex and socio-economic status.

^a^Minimum of once a week.

^b^Minimum of once a month.

^c^Minimum of once a year.

^d^Overall participation.

^e^More than 30 min per day.

^f^More than 5 h per day.

### Associations between leisure behavior and behavioral difficulties

As shown in Table 5, respondents reported significantly fewer behavioral difficulties if they engaged more frequently in family activities, meeting friends, attending a concert/opera, and sports participation (*b* ranging between –0.77 and –1.42, all *p* < 0.05). Chilling at least once per week and long screen time, in contrast, were significantly associated with more behavioral difficulties (*b* = 1.15 and 2.28, both *p* < 0.01). No significant associations were observed between the remaining leisure activities and behavioral difficulties.

**Table 5:** Association of the SDQ-score with leisure activities among healthy adolescents in the LIFE Child study cohort (*N* = 959): Results of linear mixed-effects model.

Leisure activity	SDQ-score
	*b* (95% CI)
Family time^a^	–1.32 (–1.97; –0.68)**
Meeting friends^a^	–1.19 (–1.90; –0.48)**
Chilling^a^	1.15 (0.38; 1.92)**
Café visits^b^	–0.48 (–1.14; 0.18)
Museum/Art exhibition/Cinema^b^	–0.40 (–1.19; 0.39)
Concert/Opera^c^	–1.42 (–2.17; –0.67)**
Choir/Orchestra/Theater^d^	–0.32 (–0.97; 0.34)
Voluntary work^d^	–0.42 (–1.06; 0.23)
Reading^e^	–0.47 (–1.41; 0.48)
Screen time^f^	2.28 (1.45; 3.11)**
Sports participation^d^	–0.77 (–1.44; –0.11)*

*SDQ-Score* strengths and difficulties questionnaire, *b* non-standardized regression coefficient, 95% *CI* 95% confidence interval.

*p < 0.05, **p < 0.01. All associations are adjusted for age, sex, and socioeconomic status.

^a^Minimum of once a week.

^b^Minimum of once a month.

^c^Minimum of once a year.

^d^Overall participation.

^e^More than 30 min per day.

^f^More than 5 h per day.

### Association between the leisure behavior of parents and adolescents

As presented in Table 6, adolescents reported higher participation in the described leisure activities if their parents did so, too. For all leisure activities except visiting museum/art exhibitions/cinema and sports participation, the associations were statistically significant.

**Table 6:** Associations between leisure activities of adolescents and parents among healthy adolescents in the LIFE Child cohort (*N* = 838): Results of logistic mixed-effect models.

Leisure activity	Association with parental leisure engagement
	OR (95% CI)
Family time^a^	2.51 (1.80; 3.51)**
Meeting friends^a^	2.10 (1.51; 2.92)**
Chilling^a^	1.69 (1.11; 2.57)*
Café visits^b^	2.74 (1.93; 3.88)**
Museum/Art exhibition/Cinema^b^	1.16 (0.78; 1.72)
Concert/Opera^c^	3.01 (1.75; 5.18)**
Choir/Orchestra/Theater^d^	1.56 (1.07; 2.27)*
Voluntary work^d^	1.78 (1.33; 2.40)**
Reading^e^	3.02 (1.51; 6.03)**
Screen time^f^	2.42 (1.50; 3.91)**
Sports participation^d^	2.06 (1.04; 4.09)^x^

*OR* odds ratio.

*p < 0.05, **p < 0.01, x = statistically significant before, but not after correction for multiple testing. All associations are adjusted for age, sex, and socioeconomic status.

^a^Minimum of once a week.

^b^Minimum of once a month.

^c^Minimum of once a year.

^d^Overall participation.

^e^More than 30 min per day.

^f^More than 5 h per day.

## Discussion

The purpose of this study was to investigate the associations between leisure activities and well-being in adolescents. These associations differed depending on the type of leisure behavior. Spending time with family and friends, attending cultural events, volunteering, and sports participation were associated with higher well-being, particularly better physical well-being. Activities such as chilling and media consumption, on the other hand, were associated with poorer well-being and more behavioral difficulties. Furthermore, the hypothesis that adolescents participate more in an activity when their parents do the same could largely be confirmed.

### Associations between leisure activity and sex, age and SES

As already reported in previous studies,^19,32,33^ girls reported higher participation in most leisure activities (e.g., choir/orchestra/theatre, reading) than boys. Consistent with previous findings,^19^ our analyses also revealed no significant difference in total screen time of boys and girls. However, in contrast to previous research,^19,34^ no significant association was found between sex and sports participation. This could be because we only distinguished between participation and nonparticipation in organized sports and not between intensity and frequency.

We observed that older adolescents engage more frequently in activities such as chilling and visiting a café and less frequently in family time and reading. This might be explained by the increasing need to become an independent adult and to explore one´s interests and possibilities. Furthermore, the older age group used significantly more electronic media, which confirms own previous findings.^19,23^

Adolescents from low/medium SES families were significantly less likely to participate in the observed extracurricular activities. However, they reported significantly longer screen times and more frequent chilling, supporting the current state of research.^19,23,32^ Low SES families may have neither time nor the financial or logistical means to accompany their children to extracurricular activities or to pay for their participation.

### Associations between leisure activity and well-being

In line with our hypothesis and previous research findings,^16,17,35–38^ our analyses showed that adolescents report better well-being when they engage more frequently in active leisure activities (e.g., family time, meeting friends, café visits, visiting a museum/art exhibition/cinema, visiting a concert/opera, voluntary work, sports participation).

This finding supports previous findings suggesting positive effects of volunteering^39–41^ and sports participation^2,42^ on well-being and health. However, based on previous studies,^3,42–44^ we also expected a significant association between sports participation and psychological wellbeing, which was not observed in our study. Again, this could be because we only distinguished between participation and nonparticipation in organized sports and not between intensity and frequency.

In contrast to active leisure behavior, passive leisure activities (e.g., screen time, chilling) were associated with poorer well-being. This finding strengthens the suggestion that intensive use of electronic media can have negative effects on adolescents’ mental^8,45–47^ and physical health.^10^ Given the cross-sectional design of the present (and most previous) studies in this field, the direction of effects (from leisure behavior to well-being or vice versa) remains unclear. It is also possible that well-being impacts the engagement in leisure activities. Higher well-being might motivate adolescents to be more active, while low well-being might increase rather passive behavior.

### Associations between leisure behavior of adolescents and parents

As expected and in line with previous findings,^22,23,48^ adolescents reported higher participation in the described leisure activities if their parents did so, too. This could partly be explained by the fact that parents and adolescents do certain activities together (e.g., family time, but also screen time or visiting concert/opera). Another explanation, however, is that adolescents copy their parents’ behavior or that parents motivate their children to pursue activities that they like themselves. Research is divided on the relationship between parents’ sports participation and their children’s involvement in organized sports. While some studies indicate that parents’ physical activity is associated with higher activity levels in their children,^49^ others have found no significant associations between parents’ involvement and their children’s sport participation.^50^ In line with the latter, we observed no significant association between sports participation of parents and children.

### Strengths and limitations

The strengths of this study are the variety of leisure activities that have been considered and the consideration of the parents’ leisure engagement. Similar to previous research, low SES families were underrepresented, which limits the generalizability of our results. In a more representative sample, the percentage of adolescents participating in certain leisure activities and the assessed associations (especially those between leisure activity and SES) might be different. Future research must find a way to reach more families with low SES. Another limitation of our study is that questionnaires on leisure behavior (including sports participation and media use) were no standard questionnaires. Therefore, we have no information on their validity or reliability. Additionally, we relied on self-reported frequencies of leisure activities, which might be prone to several biases. The dichotomization also limits the generalizability of our results. There might be differences within groups that cannot be addressed in this study. Finally, we only performed cross-sectional analyses that do not allow conclusions about causal relationships. We suggest conducting a longitudinal study with a larger study population in further research to be able to draw causal relationships.

### Conclusion and practical guide

This study highlights the importance of active leisure behavior (e.g., sports participation and other hobbies) for adolescents as it may improve well-being in several areas. On the other hand, passive leisure behaviors (e.g., screen time) might have negative effects on well-being.

Our findings further suggest that parents can influence their children’s well-being through their own activities.

Therefore, we need to promote not only the leisure time behavior of young people but also that of their parents and thus increase leisure time participation overall, especially in families with lower SES. Parents should be made aware that they have a role model function and that they should encourage active leisure activities for their children. In addition, adolescents could be informed in schools about the possibilities, the importance, and the potential effects of different leisure activities. At the same time, it is important to facilitate the access to active leisure activities, also for disadvantaged families, e.g., through free programs close to home.

## Data Availability

Data cannot be shared publicly because of ethical restrictions. Publishing data sets is not included in the informed consent provided by the study participants. Furthermore, the data protection concept of LIFE requests that all (external as well as internal) researchers interested in accessing the data sign a project agreement. Researchers who are interested in accessing and analyzing data collected in the LIFE Child study may contact the data use and access committee forschungsdaten@medizin.uni-leipzig.de.
